# Whole genome sequencing of peach (*Prunus persica *L.) for SNP identification and selection

**DOI:** 10.1186/1471-2164-12-569

**Published:** 2011-11-22

**Authors:** Riaz Ahmad, Dan E Parfitt, Joseph Fass, Ebenezer Ogundiwin, Amit Dhingra, Thomas M Gradziel, Dawei Lin, Nikhil A Joshi, Pedro J Martinez-Garcia, Carlos H Crisosto

**Affiliations:** 1 Department of Plant Sciences, University of California, Davis, One Shields Ave. Davis, CA 95616, USA; 2 Bioinformatics Core, University of California, Davis, CA 95616, USA; 3 Department of Horticulture and Landscape Architecture, Washington State University, Pullman, WA 99164, USA

## Abstract

**Background:**

The application of next generation sequencing technologies and bioinformatic scripts to identify high frequency SNPs distributed throughout the peach genome is described. Three peach genomes were sequenced using Roche 454 and Illumina/Solexa technologies to obtain long contigs for alignment to the draft 'Lovell' peach sequence as well as sufficient depth of coverage for 'in silico' SNP discovery.

**Description:**

The sequences were aligned to the 'Lovell' peach genome released April 01, 2010 by the International Peach Genome Initiative (IPGI). 'Dr. Davis', 'F8, 1-42' and 'Georgia Belle' were sequenced to add SNPs segregating in two breeding populations, Pop DF ('Dr. Davis' × 'F8, 1-42') and Pop DG ('Dr. Davis' × 'Georgia Belle'). Roche 454 sequencing produced 980,000 total reads with 236 Mb sequence for 'Dr. Davis' and 735,000 total reads with 172 Mb sequence for 'F8, 1-42'. 84 bp × 84 bp paired end Illumina/Solexa sequences yielded 25.5, 21.4, 25.5 million sequences for 'Dr. Davis', 'F8, 1-42' and 'Georgia Belle', respectively. BWA/SAMtools were used for alignment of raw reads and SNP detection, with custom PERL scripts for SNP filtering. Velvet's Columbus module was used for sequence assembly. Comparison of aligned and overlapping sequences from both Roche 454 and Illumina/Solexa resulted in the selection of 6654 high quality SNPs for 'Dr. Davis' vs. 'F8, 1-42' and 'Georgia Belle', distributed on eight major peach genome scaffolds as defined from the 'Lovell' assembly.

**Conclusion:**

The eight scaffolds contained about 215-225 Mb of peach genomic sequences with one SNP/~ 40,000 bases. All sequences from Roche 454 and Illumina/Solexa have been submitted to NCBI for public use in the Short Read Archive database. SNPs have been deposited in the NCBI SNP database.

## Background

Peach (*Prunus persica *L), is a member of the Rosaceae. Other important Rosaceae crop species are cherry, apricot, plum, almond, strawberry, raspberry, and rose. Peach is a model plant for the family Rosaceae due to its small genome size of ~ 230 Mb http://www.rosaceae.org/peach/genome with eight haploid chromosomes [1]. A number of molecular marker maps have been generated for *Prunus *species, some with reasonably complete coverage of all chromosomes. Eight peach maps, described by Horn et al. [2], Zhebentyayeva et al. [3], and Sosinski et al. [4], are available, all use different parents and different markers and/or marker classes. In most cases less than 220 markers have been mapped on individual peach maps, for an average marker interval of 0.82 Mb. Additional markers are needed for the next generation of mapping and gene discovery under candidate regions of interest.

Physical mapping of peach has been underway for several years. Zhebentyayeva et al. [3] constructed a BAC based physical map for peach. Sosinski et al. [4] have described the development of a draft peach genome from sequencing. The draft genome derived from the 'Lovell' haploid (peach v1.0) was released by the International Peach Genome Initiative (IPGI) at the Genome Database for Rosaceae http://www.rosaceae.org/peach/genome, also available at the Joint Genome Institute Phytozome database http://www.phytozome.org/peach.

During the last two decades DNA based molecular markers have been extensively used for evaluation of population diversity estimation, germplasm characterization, linkage and QTL analysis, gene tagging, and map-based cloning. The development of inexpensive high throughput technologies [5] for detection of Single Nucleotide Polymorphisms (SNPs) has resulted in the replacement of other DNA based marker systems as the genetic marker of choice. Large numbers of SNPs are available in most eukaryotic genomes and are found throughout the genome, providing more complete genome coverage than older marker types [6]. Expressed Sequenced Tags (ESTs) were previously the main source for SNP discovery [7], but this approach is limited to specific expressed regions of the genome conditioned by tissue type and environment. Next Generation Sequencing technologies like pyro-sequencing from Roche 454/Life Sciences, sequencing by synthesis from Illumina/Solexa and sequencing by Oligonucleotide Ligation and Detection (SOLiD) from Life Technologies, Inc. have provided rapid and inexpensive methods to sequence whole genomes and transcriptomes of individual plants in small laboratories [8]. This improved sequencing capacity can now be used to do genome-wide SNP discovery for non-model organisms [9-12].

Three parents, 'Dr. Davis' (DD), 'F8, 1-42' (F8), and 'Georgia Belle' (GB) were used to produce two breeding populations, Pop DF ('Dr. Davis' × 'F8, 1-42') and Pop DG ('Dr. Davis' × 'Georgia Belle'). The F8 parent contains an especially wide range of diversity since 'Nonpareil' almond, the old peach cultivar 'Reigels', and the dwarf peach '54P455' are part of its pedigree. It is non-melting with flesh firmness at maturity comparable to the standard canning clingstone peach cultivar, 'Dr. Davis'. Unlike standard canning clingstone peach cultivars, however, the endocarp detaches freely from the mesocarp (i.e. freestone) in F8. 'Dr. Davis' is a clingstone, non-melting, bland-flavored, non-mealy, slight-browning, yellow-flesh cultivar while 'Georgia Belle' is a freestone, melting, white-flesh cultivar with a sharp-flavor, mealy texture, and considerable flesh browning.

## Construction and content

### 454 sequencing

Nuclear DNA was isolated from 'Dr. Davis' and 'F8,1-42', with the method of Folta and Kaufman [13] to avoid organelle DNA contamination. A single run of 454 shotgun sequencing generates about 140 Mb of apple sequence (Dhingra, personal communication), and the genome size of peach is estimated at approximately 230 Mb. Two runs were conducted on each parental DNA sample for a total of four runs and approximately 1 × genome coverage for each parent using single end reads (paired end reads were not available when these runs were done). Because the coverage for these sequences was low and because the Illumina/Solexa technology became available at lower cost shortly after completion of the Roche 454 sequencing, we increased our genome coverage depth with additional Illumina/Solexa runs.

### Illumina/Solexa sequencing

We added 'Georgia Belle' to the project as the lower cost high throughput Illumina/Solexa sequencing technology became available. The GB sequence data and aligned sequence was generated only via Illumina/Solexa sequencing without addition of 454 sequence. Five ug, each, of high quality DNA of DD, F8, and GB at a concentration > 100 ng/ul, (OD 260/280 close to 1.8) in a TE ([EDTA] = 0.1 mM were isolated with the DNeasy Plant Mini kit (Qiagen, CA 91355 Valencia). DNA was converted into small fragments with the Diagenode Bioruptor (Diagenode, Denville NJ, USA) for sequencing. We quantified the library DNA with the Agilent 2100 Bioanalyzer (Agilent, Foster city, USA) which provided ng DNA/ul values, an accurate molecular weight, and the calculated molarity for each peak, while displaying the presence/absence of other unwanted library components like adapter and primer dimers. Bioanalyzer quantification indicated that the libraries generated for the three parents had a good sequence read range. SYBR green fluorescence detection was used during amplification with the library PCR sequences as an additional quality check and indicated that the libraries were of a quality suitable for sequence analysis. (The amplification efficiency of an uncharacterized library is simultaneously compared with the amplification efficiency of a previously sequenced library.).

We used the high quality libraries described above to generate paired end sequences for DD, F8, and GB. The UC Davis DNA Technology Core Facility conducted the Illumina/Solexa flow cell sequencing by running 85 cycles on the Illumina Genome Analyzer II. Each flow cell has eight lanes, corresponding to eight libraries. These can be different libraries or replicates of the same library. We used one lane to sequence the phiX174 DNA control to verify that run quality scores were being met. Each library has a particular sequencing primer used in conjunction with that library type. We used the Goat module (Firecrest v.1.8.28 and Bustard v.1.8.28 programs) of the Solexa pipeline v.0.2.2.3 for image de-convolution and quality value calculation. Parameterization was auto-generated by the pipeline. Set up configuration was used as installed by Illumina's technical staff. Quality specifications were determined by using the behavior of commercially available library DNA of phi X174. We evaluated DNA quality with the metrics: percent of the clusters that pass quality filtering, number of sequences that align to the phiX174 reference genome, and the overall percent error rate for those aligning phiX174 sequences.

### Sequences

**Figure 1:**
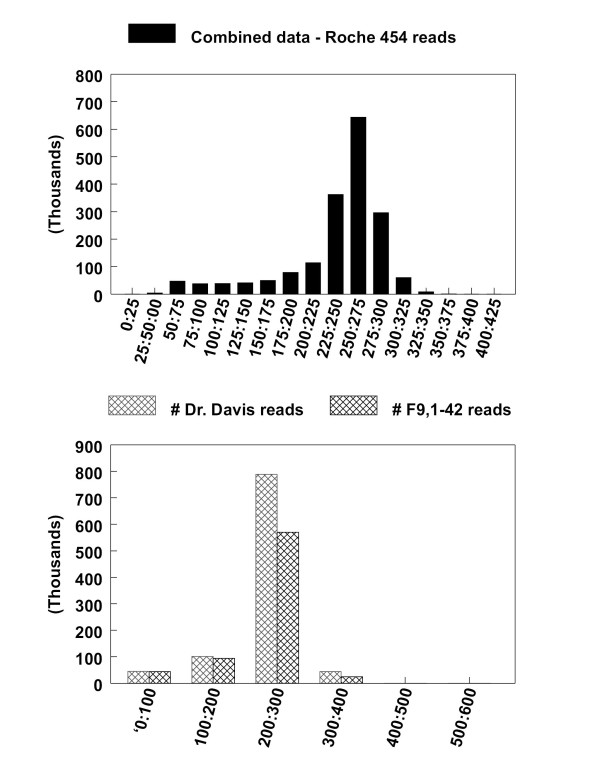
**Distributions of fragment sizes from Roche 454 sequencing, for DD and F8 combined data, and separate DD and F8 data**.

Two sequencing runs on DD and F8 using Roche 454 shotgun sequencing generated 0.408 Gb of sequence. The total number/length of sequences for DD and F8 were 980,000/236 Mb and 735,000/172 Mb, respectively. This provided an estimated 1.7 × peach genome coverage for the combined data set, appx. 1.0× for DD and 0.7× for F8, assuming a peach genome size of 230 Mb. Sequences from both runs were assembled into approximately 185,000 contigs with an average length of 258 bp (N50) via 'de novo' assembly. More than 30,000 contigs were longer than 500 bases (Figure 1) Total GC content was 38.71% (164,473,355 Mb).

Illumina/Solexa sequencing of total DNA was conducted in an 84 × 84 format that yielded pre-trimmed 25.5, 21.5 and 25.5 million read pairs for DD, F8, and GB, respectively. After post sequence trimming, ~ 4.0 Gb, ~ 3.4 Gb and ~ 3.5 Gb of nuclear and organelle sequence data were retained for DD, F8, and GB, respectively. The average length of a set of sequences (N50) was appx. 260 bp. Approximately 3.3 Gb, 2.6 Gb, 2.8 Gb of DD, F8, and GB sequence, respectively, mapped to the 'Lovell' draft genome, while 20% of the sequence did not align to 'Lovell'. After aligning the sequences to the draft 'Lovell' genome which contains only nuclear DNA, coverage depth for DD, F8, and GB were calculated to be 15.64×, 12.56×, and 13.29×, respectively, for a total coverage of 41.5× of the nuclear genome. Roche 454 and Illumina/Solexa combined pileup gave 215.2 million aligned positions (94.7%) for DD, 209.0 million positions (92.0%) for F8 and 213.0 million positions (93.7%) for GB. We detected approximately 165,000 SNPs after the Roche 454 and Illumina/Solexa sequences were combined, assembled, and aligned to 'Lovell'.

### Bioinformatic analysis

Off Line base calling was used in this analysis, (vs. standard base calling) when the run was being conducted. Following analysis of each run, the Illumina pipeline output was input into the Solexa LIMS (SLIMS) created by the UC Davis Bioinformatics Core at the Genome Center so that all the images, sequence files and experiment summaries could be examined. Custom *Perl *scripts (trim.pl, http://wiki.bioinformatics.ucdavis.edu/index.php/Trim.pl; IIIQ2SanQ.pl, http://wiki.bioinformatics.ucdavis.edu/index.php/IllQ2SanQ.pl) were used to prepare the combined Illumina/Solexa and Roche 454 sequences for alignment (format conversion, quality trimming, etc.), followed by alignment using the Burrows-Wheeler Aligner (BWA) tool [14,15]http://bio-bwa.sourceforge.net against the 'Lovell' genome (JGI's phytozome.net database). Reads were aligned to the 'Lovell' genome using BWA's long read aligner (for 454 reads) and short read aligner (for Illumina reads), at default settings. BWA finds the best positions of reads aligned to a reference sequence, and is thus distinct from an assembler, which can find overlaps between shorter sequences and use the results to generate longer contiguous sequence from which those 'de novo' short sequences might have come [14,15].

Alignments in SAM format, of the reads from the three parents, were processed using SAMtools [16]http://samtools.sourceforge.net/index.shtml to filter and report high quality SNP positions. SNP detection was performed using SAMtools' 'pileup' command at default settings appropriate for diploid organisms. SNP filtering was performed in two steps, first using SAMtools' 'varFilter' command specifying fairly permissive minimum quality cutoffs, coverage depth min/max cutoffs of 4 and 100, as well as options to disqualify SNPs that are too close to each other (as required by Illumina's GoldenGate Assay). The model used by SAMtools to call SNP positions can take heterozygosity (or ploidy in general) into account. This model is described in a paper on a previous tool from which SAMtools (and BWA) inherited code: MAQ [17]. A custom *Perl *script (Additional File 1) was developed to select positions where only one parent was polymorphic so that progenies would be heterozygous in a 1:1 ratio. Consensus sequences for the SNP positions and 60 bp on either side (121 bp total) were generated using a custom *Perl *script (SNPseqRetrieve.pl, http://wiki.bioinformatics.ucdavis.edu/index.php/SNPseqRetrieve.pl), followed by BWA alignment to align these candidate SNP sequences to the 'Lovell' sequence ftp://ftp.jgi-psf.org/pub/JGI_data/phytozome/v7.0/Ppersica/. Sequences that had any local alignment to repetitive sequence were discarded. Finally, remaining candidate SNP sequences were submitted to Illumina's Array Design Tool, which scores sequences for primer design criteria.

The 'Velvet' routine was used for reference-assisted assembly [18,19]. A recent version of 'Velvet', which includes a novel program module 'Columbus,' http://www.ebi.ac.uk/~zerbino/velvet/ was used to improve the 'de novo' assembly by utilizing alignments (via BWA) of the reads against the 'Lovell' genome. Align-able reads produce a simplified *De Bruijn *graph structure because sequence overlaps are initially restricted to the aligned region. However, reads that are dissimilar from 'Lovell' can also co-assemble, or assemble 'de novo', with the aligned reads. This simplifies the overall assembly, by reducing the complexity of the problem with information derived from the alignment. This approach provided a good total assembly, but the resulting sequences remained fragmented. The fragmented contigs were anchored to the 'Lovell' genome, to develop scaffolds based on the 'Lovell' assembly, by using the MAUVE aligner [20]http://asap.ahabs.wisc.edu/mauve/ and custom PERL scripts. In addition to the contigs anchored to the 'Lovell' scaffolds, we obtained a set of contigs that could not be aligned to 'Lovell' due to lack of homology. After creating a SAM-format alignment of the parent sequences referenced to 'Lovell', SNP positions and indels were located with SAMtools. SAMtools incorporates the MAQ model for new consensus calling. A custom platform was developed in GBrowse for deposition of SNP, indel, and sequence data.

### SNP selection

The ~165,000 SNPs were passed through a series of bioinformatics filters. The first filter selected SNPs so that candidates have no neighboring gaps or SNPs within 60 bp on either side of the target SNP. The second filter selected SNPs for which only one parent is polymorphic (permitting 1:1 segregation in the progeny), with SNP quality Q > 100 (phred-like score). The third filter selected SNPs which are present in 10 or more reads and resulted in the retention of ~9000 SNPs. The fourth filter removed SNPs in repeat-regions using Repeat Masker. After running Repeat Masker, 6654 SNPs were retained. The selected 6654 SNPs all have SNP qualities of at least 100 (1 in 1000 chance of an erroneous SNP call). The highest quality SNPs, Q > 200, were distributed evenly across the major scaffolds (Table 1) as defined by the 'Lovell' genome while scaffolds 7 and 8 had a higher concentration of SNPs of Q > 100 than the other scaffolds. The 8 major scaffolds contained ~225 million nucleotide bases of peach genome with approximately one SNP/40,000 nucleotides (Std. Dev. 22,441 nucleotides, CV = 55%).

**Table 1:** Peach genome scaffold information with the number of SNP's detected on each scaffold.

Scaffold ID	#SNPs w/quality > 100	#SNPs w/quality > 200	Scaffold length (nt)	Scaffold length (nt)/SNP > 100	Scaffold length (nt)/SNP > 200
scaffold 1	1267	716	46,877,626	36,999	65,472
scaffold 2	1205	412	26,807,724	22,247	65,067
scaffold 3	499	241	22,025,550	44,140	91,392
scaffold 4	412	227	30,528,727	74,099	134,488
scaffold 5	698	357	18,502,877	26,508	51,829
scaffold 6	392	217	28,902,582	73,731	133,192
scaffold 7	862	414	22,790,193	26,39	55,049
scaffold 8	1266	459	21,829,753	17,43	47,559

scaffold 9	9	4	2,126,789	236,310	531,697
scaffold 10	0	0	851,981	na	na
scaffold 11	1	8	736,058	736,058	92,007
scaffold 12	27	8	675,284	25,011	84,411
scaffold 13	0	0	670,721	na	na
scaffold 14	1	0	575,512	575,512	na
scaffold 15	14	8	516,056	3861	64,507
scaffold 16	1	1	390,024	390,024	390,024
scaffold 17	0	0	370,749	na	na
scaffold 18	0	0	333,953	na	na
scaffold 22	0	0	167,479	na	na
scaffold 23	0	0	69,963	na	na

Total	6654	3065	225,749,601	ave. = 40,176	ave. = 80,506

### Data Release

Scripts developed for this project are available to researchers at http://wiki.bioinformatics.ucdavis.edu/index.php/Trim.pl and http://wiki.bioinformatics.ucdavis.edu/index.php/IllQ2SanQ.pl. All sequences from Roche 454 and Illumina/Solexa have been submitted to NCBI for public use in the Short Read Archive database as 'objects' (search string "UC Davis peach"). SRA accession numbers are SRP003772 ('Dr. Davis'), SRP003847 ('F8, 1-42'), and SRP003848 ('Georgia Belle') in http://www.ncbi.nlm.nih.gov/sra?term=Sequence Read Archive. All SNPs have been deposited in the NCBI SNP database (dbSNP) at http://www.ncbi.nlm.nih.gov/SNP/snp_viewTable.cgi?handle=UCDAVISBIOINFO.

The SNPs are in the range [NCBI-dbSNP:275372743 to NCBI-dbSNP:275395485].

## Utility

The SNP data sets described in this submission were designed to be used as a resource for future map development and QTL analysis of quality genes in progeny from crosses developed by the University of California, Davis peach and almond breeding programs. One of our objectives was to locate a SNP approximately every 40,000 bases on the physical peach map to assist with the characterization of functional genes after mapping and QTL analysis. Many of the SNPs may be useful in other peach improvement programs with similar objectives and are provided as a community resource without restriction.

80% of the Illumina/Solexa raw reads from the present analysis were useable. Inclusion of the Roche 454 sequence greatly improved our alignment to the 'Lovell' sequence because Roche 454 sequences aligned to genome areas that Illumina/Solexa sequences did not uniquely or usefully align to. 93.4% of the combined pileup of Roche 454 and Illumina/Solexa reads aligned to the reference genome. For comparison, Harismendy et al. [21] found that on average, 55% of the Illumina Genome Analyzer reads passed quality filters, of which approximately 77% aligned to the reference sequence. For ABI SOLiD, approximately 35% of the reads passed quality filters, and subsequently 96% of the filtered reads aligned to the reference sequence. Thus, only 43% and 34% of their Illumina Genome Analyzer and ABI SOLiD raw reads, respectively, were useable. Removal of repeat regions before SNP selection was an important step in the current analysis, because according to Harismendy et al. [21] more than 72% of false positive and negative calls were associated with repetitive elements, homopolymers ≥ 6 bases long, or the presence of an indel within 30 bp of the target SNP. Brockman et al. [22] suggested that more than 30× coverage should be used for finding SNPs in repetitive or highly polymorphic regions. Dohm et al. [23] reported that using a combination of single and paired end reads is a good approach for many assembly projects, while Bentley et al. [24] suggested that longer single reads are better for SNP discovery. Sequences from regions of highly repeated DNA are of poor quality and the paired end strategy helped to resolve differences among repeat regions and lowered the sequence error rate. Sufficient 60 bp flanking sequence on either side of the SNPs was obtained from paired end reads to effectively design high quality primers for SNP identification. The amount of homopolymer miscounting errors (i.e, "TTTTTTT" from the Roche 454 reads in the actual sequence as 5 or 7 T's instead of 6) and degraded sequence quality at the 3' end of Illumina/Solexa sequences was substantially reduced by trimming the ends and by the paired end sequencing strategy. The homopolymer problem was dealt with by filtering out SNPs that appeared near gaps in the alignment, since homopolymer errors often cause these gaps (and artificial SNPs) when the gaps incorrectly offset neighboring sequence.

The 6654 SNPs from this study were validated by selecting a subset of 1536 SNPs, spaced at reasonably even intervals along the aligned genomes, and mapping them to Pop DF and Pop DG. 1211 SNPs were mapped in both populations. 304, 385, and 549 unique map positions (either one or more SNPs located at those positions) were obtained for Pop DF, Pop DG, and the consensus map, respectively, and are reported in a separate manuscript.

## Discussion

The total base output from each lane of the Illumina/Solexa sequencing protocol depends on the number of cycles used during the run, and whether or not the reads are paired end reads. For example, a 40 cycle single read run with 24 million good reads will give 960 Mb bases/lane, while an 85 cycle run of paired end reads will provide nearly 4 Gb. These results are better than those of Harismendy et al. [21,13], where they obtained an average of 49,000 reads per sample for the Roche 454 platform with an average length of 245 bp while their Illumina Genome Analyzer runs generated an average of 5.9 million reads, each 36 bases in length per sample, and ABI SOLiD resulted in approximately 19.7 million reads, each 35 bases in length per sample. This improvement reflects the extremely rapid development of next generation sequencers.

Combining the Roche 454 long reads of ~ 250 bp and ~ 84 bp read length from the Illumina/Solexa method improved sequence alignments. Early reads from Illumina/Solexa rarely exceeded 36 bp, but at the time that this project was conducted read lengths of greater than 84 bp were being generated. (More than 80% of the reads from Illumina/Solexa in the present study were ~ 84 bp in length.) The consensus view from many of the large sequencing centers (C. Nicolet, personal communication), suggests 85 bp runs are the best compromise between longest read length and lowest error. Sequences of this size are suitable for SNP discovery applications, for transcriptome analysis, and for bacterial genome analyses [22]. The alignment algorithms released by Illumina as part of their pipeline are becoming increasingly sophisticated and have more functionality for identification of indels, splice junctions, tag counts, etc than did earlier versions. Sequences can be assembled 'de novo' or aligned against a completed reference sequence. The latter approach is more efficient.

## Conclusions

The pyro-sequencing technologies of Roche 454 and Illumina/Solexa were used to generate deep coverage genome sequence (43.2× combined Roche and Illumina/Solexa) from the three peach genomes at a reasonable cost. Customized *Perl *scripts were developed to analyze the sequence information, with the recently released Velvet software including the Columbus module for sequence assembly, to select an initial set of ~6,000 candidate SNPs from those sequences in a cost effective manner.

The peach sequence data was used to identify SNPs for subsequent mapping and use in breeding strategies. New *Perl *scripts were developed to evaluate and refine SNP populations and to identify useable SNPs for subsequent mapping. The 11.3 Gb of submitted peach sequence data sets and the 6654 SNP data set are a resource that can be used by the peach breeding community to mark genes of interest in different breeding programs. However, some of these SNPs may not be present for all combinations of peach parents. Additional SNP discovery using the technologies described in this paper will be needed to develop SNP panels optimized for specific parents and cross combinations. The 6654 SNPs discovered in the present study and their distribution on all scaffolds with ~1 SNP/40,000 nucleotide bases will cover the peach genome sufficiently to permit tight SNP linkage to functional genes for breeding and/or functional gene characterization as well as marking QTLs for breeding.

## Abbreviations

Peach parents are denoted as DD: 'Dr. Davis'; F8: 'F8, 1-42'; GB: 'Georgia Belle'. Progeny populations from crosses among the parents are Pop DF: 'Dr. Davis' × 'F8, 1-42'; Pop DG: 'Dr. Davis' × 'Georgia Belle'. NCBI is the National Center for Biotechnology Information, while SNPs are single nucleotide polymorphisms.

## Authors' contributions

RA collected samples, organized the plant materials, coordinated data collection with JF and wrote first draft of paper. DEP planned project with EO and CHC, coordinated activities of RA, JF, and DL, revised and edited grant proposal and paper, and served as communicating author. EO planned project with CHC and DEP, wrote draft grant proposal. JF developed Perl scripts, analyzed sequences and selected SNPs. AD provided 454 data, TMG provided plant materials. DL was involved in planning the analysis of sequence data. NAJ conducted data analysis for JF. PMG evaluated SNPs after SNP selection. CHC planned project with DEP and EO, obtained the extramural funding for the project. All authors read and approved the manuscript.

## Supplementary Material

Additional file 1 **Script for SNP selection from polymorphic parents**. Custom script for selecting SNPs from polymorphic parents in crosses used to identify SNPs that will produce 1:1 segregations in mapping populations. Click here for file
