# Supplementary material for: Whole genome sequencing of peach (Prunus persica L.) for SNP identification and selection
Source: BMC Genomics. 2011 Nov 22;12:569. doi: 10.1186/1471-2164-12-569 (PMC3253712; doi:10.1186/1471-2164-12-569)
Supplement: Additional file 1 — Script for SNP selection from polymorphic parents. Custom script for selecting SNPs from polymorphic parents in crosses used to identify SNPs that will produce 1:1 segregations in mapping populations. [file 1471-2164-12-569-S1.DOCX]

**Additional Files**

**Additional File 1**

**Title:** Script for SNP selection from polymorphic parents.

**Description:** Custom script for selecting SNPs from polymorphic parents in crosses used to identify SNPs that will produce 1:1 segregations in mapping populations.

#!/usr/bin/perl

# AUTHOR: Joseph Fass

# LAST REVISED: September 2010

#

# The Bioinformatics Core at UC Davis Genome Center

# http://bioinformatics.ucdavis.edu

# Copyright (c) 2010 The Regents of University of California, Davis Campus.

# All rights reserved.

#

# one-off script to print pileup lines based on intersections or unions of two 'samtools pileup -cv' outputs ...

# read master SNPs list

open FID, "<combinedSNPs.txt";

while ($line = <FID>) {@line = split /\t/, $line;

$all{$line[0]}{$line[1]} = $line;}

close FID;

# read DD subset

open FID, "<combinedSNPs.DDsubset.txt";

while ($line = <FID>) {@line = split /\t/, $line;

$DD{$line[0]}{$line[1]} = $line; # "*" bases (insertion/deletions) will overwrite previous line, which is OK ... it disqualifies position}

close FID;

# read F8 subset

open FID, "<combinedSNPs.F8subset.txt";

while ($line = <FID>) {@line = split /\t/, $line;

$F8{$line[0]}{$line[1]} = $line; # "*" bases (insertion/deletions) will overwrite previous line, which is OK ... it disqualifies position}

close FID;

# now, iterate through key pairs and find SNPS that only exist in one parent

foreach $scaff (keys %all) {

LOOP: foreach $pos (keys %{$all{$scaff}}) {

@allLine = split /\t/, $all{$scaff}{$pos};

@DD_Line = split /\t/, $DD{$scaff}{$pos};

@F8_Line = split /\t/, $F8{$scaff}{$pos};

next LOOP if ($DD_Line[2] eq "*" or $F8_Line[2] eq "*"); # I/D disqualifies position

next LOOP if ($allLine[3] =~ m/[ATCG]/); # non-ambigious consensus disqualifies

next LOOP if ($DD_Line[8] =~ m/[ATCG]/ and $F8_Line[8] =~ m/[ATCG]/); # non-[.,] in *both* pileups disqualifies position

next LOOP if (!( ($DD_Line[5]<1 and $F8_Line[5]>100) or ($DD_Line[5]>100 and $F8_Line[5]<1) )); # must have one SNP (quality)

next LOOP if ($DD_Line[7]<10 or $F8_Line[7]<10); # must have depth >= 10 in both

$allNT = $allLine[3]; # consensus from combined mapping

$DD_NT = $DD_Line[3]; # consensus from DD mapping

$F8_NT = $F8_Line[3]; # consunsus from F8 mapping

$refNT = $allLine[2]; # Lovell reference base

$maxSNPq = $DD_Line[5]; # DD SNP quality

if ($F8_Line[5] > $maxSNPq) { $maxSNPq = $F8_Line[5] } # calculate max SNP quality score

# check that one parent (only) has same SNP as combined predicted SNP

# if ($DD_NT ne $F8_NT and ($allNT eq $DD_NT or $allNT eq $F8_NT)) { # for either parent hetero

if ($DD_NT ne $F8_NT and $allNT eq $DD_NT) { # for DD hetero's only

$DD_Line[8] =~ s/\^.//g; # squeeze out read start and quality characters

$DD_Line[8] =~ s/\$//g; # squeeze out read end characters

$F8_Line[8] =~ s/\^.//g; # squeeze out read start and quality characters

$F8_Line[8] =~ s/\$//g; # squeeze out read end characters

print join("\t",@allLine[0..3])."\t".$maxSNPq."\t".$DD_Line[8]."\t".$F8_Line[8]."\n"; } } }
